# Reduced work/academic performance and quality of life in patients with allergic rhinitis and impact of allergen immunotherapy

**DOI:** 10.1186/s13223-016-0146-9

**Published:** 2016-08-11

**Authors:** A. Roger, E. Arcalá Campillo, M. C. Torres, C. Millan, I. Jáuregui, E. Mohedano, S. Liñan, P. Verdu, N. Rubira, M. Santaolalla, P. González, A. Orovitg, E. Villarrubia

**Affiliations:** 1 Allergy Unit, Germans Trias i Pujol Hospital, Badalona, Spain; 2 Alergólogo. Consulta Privada, Jaén, Spain; 3 Unidad de Alergia, Hospital Casa de Salud, Valencia, Spain; 4 Unidad de Alergia, Hospital Jerez de la Frontera, Jerez de la Frontera, Spain; 5 Servicio de Alergia, Hospital Universitario de Basurto, Bilbao, Spain; 6 Servicio de Alergia, Hospital Universitario de Fuenlabrada, Fuenlabrada, Spain; 7 Neumología Pediátrica, Hospital de Nens de Barcelona, Barcelona, Spain; 8 Servicio de Alergia, Hospital Universitario de Gran Canaria Dr. Negrín, Las Palmas de Gran Canaria, Spain; 9 Unidad de Alergia, Centro Sanitario C Mora, Sant Cugat, Barcelona, Spain; 10 Servicio de Alergia, Hospital Universitario de Sanchinarro, Madrid, Spain; 11 Servicio de Alergia, Hospital General Universitario de Alicante, Alicante, Spain; 12 Unidad de Alergia, Hospital Viamed Santa Angela de la Cruz, Seville, Spain; 13 Health Outcomes Research Department, 3D Health Research, Balmes 152 6º 2ª, 08008 Barcelona, Spain

**Keywords:** Allergic rhinitis, Productivity, Daily activities, Quality of life, Allergen immunotherapy

## Abstract

**Background:**

Allergic rhinitis (AR) is characterised by burdensome nasal and/or ocular symptoms. This inflammatory disease can be debilitating and thus result in considerable health-related and economic consequences.

**Methods:**

In a cross-sectional study, adult subjects with AR (N = 683) completed three allergy-specific questionnaires that assessed the impact of AR on the work/academic performance, daily activities, health-related quality of life (HRQOL), and satisfaction with allergen immunotherapy (AIT). Regression analyses were used to examine the associations between several clinical variables and the patient-reported outcomes.

**Results:**

Total loss of productivity was 21.0 and 21.2 % for employed and student patients, respectively, whereas the impairment of daily activities was 22.0 %. The mean overall HRQOL score was 1.94 ± 1.29 (on the scale of 0–6 points). Global score for satisfaction with AIT was 65.5 ± 24.8 (on a 0–100 scale). Simple regression analysis found statistically significant associations between loss of work and academic productivity, impairment of daily activities and the type and severity of AR. AIT was a protective factor. The persistent and more severe types of AR and lack of AIT contributed to the worsening of HRQOL.

**Conclusions:**

AR (the persistent and more severe form of the disease) has an impact on functional characteristics of adult patients in Spain. AIT might reduce the effect of this disease on the work/academic performance and HRQOL.

*Trial registration* Retrospectively registered

## Background

Allergic rhinitis (AR) affects more than 400 million people worldwide, with high prevalence recorded in the developed countries [1, 2]. It is characterized primarily by nasal symptoms such as sneezing, itching, rhinorrhea, nasal congestion, and post-nasal drip. Loss of taste and smell, allodynia, or mouth breathing and snoring due to nasal congestion may also occur. The bothersome nature of AR symptoms can severely affect daily activities such as the ability to work [3–5], examination performance [6], quality of life [7], and psychosocial well-being [8].

AR is also associated with substantial economic costs [9, 10]. Indirect costs of work and school absenteeism due to AR have been estimated to be higher than those caused by diabetes, migraine, anxiety, or asthma [11]. Moreover, when the patients attend their workplaces, the symptoms lead to reduced productivity, a major problem known as “presenteeism” [12]. The studies of the socioeconomic burden of productivity loss have shown that AR and depression are of some of the most frequent causes of absenteeism, particularly during spring season [13]. The understanding of the social impact is important; a relationship between treatment adherence, health-related quality of life (HRQOL), and performance has been already demonstrated in pathologies such as asthma [14].

In Spain, a previous study estimated direct and indirect costs of AR, showing the important economic burden of the disease [15]. The work of de la Hoz Caballer et al. [5] compared the effect of AR on HRQOL and work performance with the impact of other prevalent diseases such as hypertension, diabetes mellitus type II, and symptomatic depression. In the present study, we conducted an extensive examination of the impact of AR on functional characteristics of the patients (work/academic productivity and daily activities) and HRQOL, and assessed their satisfaction with the treatment. Several factors (patient- and disease-related characteristics, the type of treatment) potentially associated with impaired performance, HRQOL, or satisfaction with the treatment, were also evaluated.

## Methods

The study was a cross-sectional, observational study conducted in allergy departments in Spain from May 2011 to October 2012. The study was approved by the Institutional Ethics Committee of the Hospital German Trias i Pujol (Badalona, Spain) and was conducted in accordance with the principles of the Declaration of Helsinki. Before participation, all patients had signed the informed consent form.

**Fig. 1:**
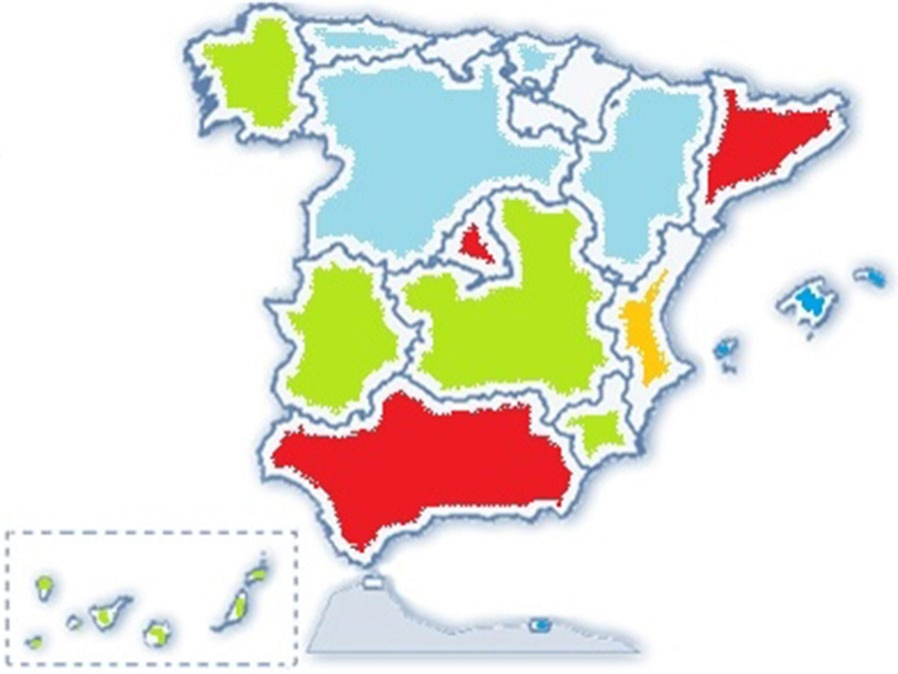
Distribution of the recruiting regions involved in the study to cover the geographical area of Spain. The map shows autonomus communities from Spain. *Blue colour* means ≤5 recruiting investigators involved; *green* 5–10 investigators; *orange* 10–15 investigators; and *red* ≥15 investigators

A number of physicians from different regions were selected to cover the geographical area of Spain (Fig. 1). Each physician included 6–7 consecutive patients throughout the four seasons of the year, preferably in a uniform manner (i.e. 2-2-1-1; 1-2-2-1; 1-1-2-2; 2-1-1-2). Patients were over 18 years old, experiencing AR (according to ARIA and Valero criteria [1, 16]), diagnosed by a prick test or specific IgE, and poorly controlled (may be also with symptomatic treatment). The patients had a stable job or pursued an academic activity. Any comorbidity that could affect work/study performance according to the physician’s criteria was an exclusion criterion. Patients participating in other clinical study or unable to understand or complete the questionnaires were also excluded.

The study data collection was performed during a single visit. Physicians completed a patient record form for each patient. The record included demographic characteristics, the information on the years since diagnosis of the disease, allergens/factors inducing nasal symptoms, disease severity, number of visits to the specialist in the last 12 months, symptoms and comorbidities (asthma, conjunctivitis, atopic dermatitis, etc.), symptomatic treatment for AR, and the use of allergen immunotherapy (AIT).

### Patient-reported health outcomes

Patients were invited to complete several self-report questionnaires. To evaluate the burden of illness, the Work Productivity and Activity Impairment Questionnaire and Classroom Impairment Questions: Allergy Specific (WPAI + CIQ:AS) were used [17]. The WPAI-CIQ-AS is a 9-item, patient-reported questionnaire. The patients reported the work time or academic classes lost due to the allergies. They self-assessed the impact of allergies on the performance in the workplace, at school, or during university classes. The patients also described the effect of allergies on other daily activities; they were asked to recall such activities during the previous 7 days. Outcomes were expressed as impairment percentages, with higher scores indicating greater impairment and reduced productivity.

The HRQOL assessment was performed using the ESPRINT-15 tool, the short form of the Spanish questionnaire specifically designed for AR patients [18, 19]. This questionnaire contains 15 items within the following dimensions: symptoms (5 items), daily activities (3 items), sleep (3 items), psychological impact (3 items), and general health (1 item). The overall score and the score for each dimension were registered, on a scale from 0 (no impact on HRQOL) to 6 (maximum impact on HRQOL).

The Satisfaction Scale for Patients Receiving Allergen Immunotherapy (ESPIA; from Spanish, “Escala de Satisfacción de Pacientes en tratatamiento con Inmunoterapia con Alérgenos”) questionnaire [20] was used to determine the satisfaction of patients receiving AIT treatment. The questionnaire consists of 16 items distributed among four dimensions: perceived efficacy, activities and environment, cost–benefit balance, and general satisfaction. The questionnaire scores (overall and for the four dimensions) are obtained by transforming the sum of its items to a 0–100-point scale, with low scores indicating low levels of satisfaction.

### Statistical analysis

We performed data management and statistical analysis using the SPSS software package (version 14; SPSS Inc. Chicago, IL, USA). A descriptive analysis was conducted, describing categorical variables using numbers and percentages, and quantitative variables using means and standard deviation (SD).

For the association of quantitative variables, the Kruskal–Wallis and Mann–Whitney tests were used. We determined predictive factors for impaired productivity and quality of life by using linear regression analysis. The dependent variables were the scores in the patient questionnaires. The multivariable model was developed, based on a backwards selection from the variables with significance in bivariate analysis. All statistical tests were considered significant at P < 0.05.

## Results

### Patient demography and clinical description

The study enrolled 683 patients recruited by 144 physicians. The patient demographics are shown in Table 1. Overall, 332 (48.6 %) were men and the mean age was 33.2 ± 10.3 years. The majority of patients (n = 525, 78.8 %) were employed or self-employed; 137 (21.1 %) were full-time students and 4 (0.6 %) were studying and working.

**Table 1:** Sociodemographic characteristics of patients

	N = 683
Sex (males), N (%)	332 (48.6)
Age (years), mean ± SD	33.2 ± 10.3
Educational level, N (%)	
Basic literacy	9 (1.3)
Primary level	73 (10.7)
Secondary level	289 (42.3)
Completed university	300 (43.9)
Employment status, N (%)	
Employed and/or self-employed	525 (78.8)
Full-time student	137 (20.6)
Student and employee	4 (0.6)

Clinical information is presented in Table 2. Mean time since rhinitis diagnosis was 10.8 ± 8.8 years. In most cases, the diagnosis of allergy was conducted using skin-prick tests (98.8 %) and/or serum allergen-specific IgE (62.8 %). The most common etiological allergens were pollens only in 48.1 %, followed by dust mites only in 29.1 % and both allergens pollen and mites in 20.5 %. The frequency of intermittent and persistent AR was 29.4 and 70.6 %, respectively. According to ARIA criteria, 92 (13.5 %) patients had mild AR and 591 (86.5 %) had moderate/severe AR, whereas Valero’s criteria classified 92 (13.5 %) as mild, 380 (55.6 %) as moderate, and 211 (30.9 %) as severe. In the preceding 12 months, the mean number of control visits to the allergist was 3.0 ± 2.6. The majority of patients (486, 71.2 %) were undergoing pharmacological treatment for AR symptoms. On the consultation day, 403 (59.0 %) and 61 (8.9 %) patients were receiving oral and/or topical antihistamines, respectively; 269 patients (39.4 %) were receiving nasal corticosteroids. Thirteen patients (1.9 %) were undergoing oral corticosteroid treatment and 47 patients (6.9 %) were being treated with antileukotrienes. From the large proportion of patients under AIT (508, 76.2 %), 304 (60.7 %) were given subcutaneous immunotherapy (SCIT) and 194 (38.3 %) were given sublingual immunotherapy (SLIT). Most of these patients stated that after the initiation of AIT they had used less medication (77.4 %) and had fewer symptoms (81.1 %).

**Table 2:** Clinical and disease information

	N = 683
AR duration (years), mean ± SD	10.8 ± 8.8
Etiologic allergen, N (%)	
Dust mite	199 (30.0)
Pollen	325 (48.9)
Dust mite and pollen	140 (21.1)
Type of AR, N (%)	
Intermittent	200 (29.4)
Persistent	480 (70.6)
ARIA Severity of AR, N (%)	
Mild	92 (13.5)
Moderate/severe	591 (86.5)
VALERO severity of AR, N (%)	
Mild	92 (13.5)
Moderate	380 (55.6)
Severe	211 (30.9)
Current pharmacological treatment, N (%)	
No	197 (28.8)
Yes	486 (71.2)
Current allergen immunotherapy, N (%)	
No	159 (23.8)
Yes	508 (76.2)
SCIT	304 (60.7)
SLIT	194 (38.7)
Both	3 (0.6)
Time from current AIT initiation (months), mean ± SD	12.8 ± 14.2
Current use of medication versus before AIT initiation, N (%)	
More medication	10 (1.9)
The same medication	112 (20.7)
Less medication	418 (77.4)
Current level of symptoms versus before AIT initiation, N (%)	
More symptoms	10 (1.9)
The same level of symptoms	92 (17.1)
Less symptoms	437 (81.1)

*AIT* allergen immunotherapy, *AR* allergy rhinitis, *SCIT* subcutaneous immunotherapy, *SD* standard deviation, *SLIT* sublingual immunotherapy

### Patient-reported measures

Mean WPAI + CIQ:AS scores were similar for the employed and the students, with the loss of productivity of 21.0 and 21.2 %, respectively. Overall mean value for the impairment of daily life activities was 22.0 %. The mean overall HRQOL score assessed using the ESPRINT-15 tool was 1.94 ± 1.29 (scale of 0–5.8 points). The symptom dimension had a high score (2.21 ± 1.38), whereas daily activities were less affected (1.67 ± 1.47). The global score in the ESPIA questionnaire filled by the patients under AIT (n = 508) was 65.5 ± 24.8 (on a 0–100 scale). Overall satisfaction with the treatment had the highest score (73.4 ± 25.7), while the score for the activity and environment dimension was the lowest (61.8 ± 27.3).

### Factors associated with impaired productivity, HRQOL, and satisfaction with AIT

Initially in the bivariate analysis, several clinical parameters were identified as potential factors significantly associated with impaired work/academic productivity and daily activities (Table 3): age, sex, education level, duration of AR, number of visits, type of AR, AR severity, and concurrent AIT.

**Table 3:** Factors potentially associated with impairment in main activity performance (WPAI + CIQ:AS) according to the bivariate and simple regression models

Factors	Dimensions of the main activity performance (WPAI+CIQ:AS)														
	Work productivity					Academic productivity					Daily activities				
	n	Bivariate analysis			Simple regression	n	Bivariate analysis			Simple regression	n	Bivariate analysis			Simple regression
		Mean score	SD	P value*	Beta coefficient		Mean score	SD	P value*	Beta coefficient		Mean score	SD	P value*	Beta coefficient
Age (years)															
<35	240	22.75	25.53	*0.078*	*Excluded*	144	23.03	26.89	*0.022*	*−0.464*	371	23.29	24.19	0.106	
≥35	237	19.18	23.61			27	11.38	19.49			263	20.27	22.86		
Sex															
Male	248	18.65	23.56	*0.022*	*Excluded*	68	19.07	25.17	0.361		305	19.34	22.44	*0.004*	*3.415*
Female	229	23.49	25.57			103	22.59	26.82			329	24.53	24.54		
Educational level															
Basic literacy	6	32.78	33.96	*0.044*	*−5.951*	2	20	28.28	0.998		8	20	19.27	0.297	
Primary level	48	28.14	26.9			6	20.07	29.41			63	25.71	23.94		
Secondary level	188	22.34	25.53			90	20.73	25.5			270	22.33	23.55		
Completed university	226	18.13	22.66			70	20.57	25.82			284	20.77	23.48		
Duration of AR (years)															
< 9	230	20.05	24.01	0.656		108	21.88	26.99	0.589		330	20.09	22.7	0.061	*Excluded*
≥ 9	243	21.58	25.07			62	19.84	25.01			300	24.03	24.52		
Number of visits to allergist															
<3	263	23.17	24.76	*0.009*	*Excluded*	102	23.14	26.46	0.231		357	24.06	23.81	*0.005*	*Excluded*
≥3	209	18.46	24.46			68	18.57	25.72			270	19.48	23.35		
Type of AR															
Intermittent	133	11.78	18.36	*0.000*	*5.960*	53	10.09	17.05	*0.000*	*17.609*	182	12.58	16.4	*0.000*	*9.127*
Persistent	344	24.53	25.83			116	26.45	28.13			450	25.91	25.09		
VALERO severity															
Mild	63	9.8	17.86	*0.000*	*8.359*	19	10.35	19.31	*0.003*	*Excluded*	84	8.69	14.29	*0.000*	*5.656*
Moderate	262	17.92	21.56			110	19.26	25.13			353	21.22	22.28		
Severe	152	30.86	28.5			42	31.13	28.8			197	29.19	26.52		
Current AIT															
No	110	30.6	25.7	*0.000*	*−9.907*	36	31.94	28.34	*0.002*	*−12.421*	141	35.67	25.17	*0.000*	*−15.575*
Yes	357	18	23.58			132	18.43	25.08			480	17.92	21.76		

“Excluded” are the factors discarded by the regression model as potentially associated with productivity impairment.Mean overall scores of the WPAI + CIQ:AS questionnaire are expressed as percentages of impairment and productivity loss

Significance values are in italics

*AIT* allergen immunotherapy, *AR* allergic rhinitis, *SD* standard deviation, *WPAI* + *CIQ:AS* Work Productivity and Activity Impairment questionnaire plus Classroom Impairment Questions: Allergy Specific

* P-value for the bivariate analysis was calculated using Mann–Whitney or Kruskal–Wallis tests for independent samples, with a confidence interval of 95 %

Simple regression analysis confirmed that work productivity was associated with the education level, with more education associated with less disease impact, type and severity of AR (persistent and more severe AR associated with more impairment), and current AIT (less impact with AIT). Academic productivity was associated with age (older people had lower impaiment), type of AR (persistent AR affected more), and current AIT (less impact with AIT). Impact on daily activities was associated with sex (female patients more affected), type and severity of AR (persistent and severe forms had a greater impact), and current AIT (less with AIT).

 As shown in Table 4, persistent and more severe AR and absence of current AIT all contributed to worse HRQOL in both employed and student patients.

**Table 4:** Factors potentially associated with impairment in health-related quality of life (ESPRINT-15) according to the bivariate and simple regression models

Factors	Health-related quality of life (ESPRINT-15)									
	Adults working					Adults studying				
	n	Bivariate analysis			Simple regression	n	Bivariate analysis			Simple regression
		Mean score	SD	P value*	Beta coefficient		Mean score	SD	P value*	Beta coefficient
Sex										
Male	259	1.79	1.22	*0.002*	*0.276*	57	1.81	1.2	0.564	
Female	257	2.18	1.38			77	1.94	1.15		
Number of visits to allergist										
<3	293	2.07	1.34	0.069		82	2.03	1.26	0.081	*−0.048*
≥3	218	1.87	1.28			51	1.62	0.98		
Type of AR										
Intermittent	140	1.37	1.06	*0.000*	*0.333*	46	1.42	1.04	*0.001*	*0.461*
Persistent	376	2.21	1.33			86	2.14	1.17		
Mild	70	1.04	0.87			16	0.93	0.85		
VALERO severity										
Moderate	282	1.86	1.14	*0.000*	*8.359*	80	1.84	1.05	*0.000*	*0.438*
Severe	164	2.59	1.46			38	2.37	1.27		
Current AIT										
No	122	2.73	1.23	*0.000*	*−0.813*	29	2.87	1.13	*0.000*	*−1.113*
Yes	382	1.74	1.25			103	1.6	1.03		

“Excluded” are the factors discarded by the regression model as potentially associated with productivity impairment

A mean overall score of the ESPRINT-15 questionnaire on the scale of 0 (no impact on HRQOL) to 6 (maximum impact on HRQOL)

Significance values are in italics

*AIT* allergen immunotherapy, *AR* allergic rhinitis, *SD* standard deviation

* P-value for the bivariate analysis was calculated using Mann–Whitney or Kruskal–Wallis tests for independent samples, with a confidence interval of 95 %

For the ESPIA scores, employed patients with persistent and more severe AR were less satisfied with their AIT, while being allergic to both dust mite and pollen and suffering intermittent AR were associated with higher scores in student patients. There was no significant difference between satisfaction with SCIT and sublingual immunotherapy (SLIT) (Table 5).

**Table 5:** Factors potentially associated with satisfaction with AIT (ESPIA) according to the bivariate and simple regression models

Factors	Satisfaction with AIT (ESPIA)									
	Adults working					Adults studying				
	n	Bivariate analysis			Simple Regression	n	Bivariate analysis			Simple regression#
		Mean score	SD	P value*	Beta coefficient		Mean score	SD	P value*	Beta coefficient
Age (years)										
<35	180	63.01	25.64	*0.040*	*Excluded*	96	65.43	24.3	0.495	*Excluded*
≥35	206	68.08	24.44			6	62.19	17.38		
Sex										
Male	200	66.18	26.49	0.347		45	62.12	25.89	0.35	*8.491*
Female	186	65.21	23.58			57	67.71	22.11		
Educational level										
Basic literacy	6	69.79	25.52	0.924		2	42.19	26.52	0.167	*Excluded*
Primary level	51	64.65	24.38			5	44.06	37.41		
Secondary level	139	65.19	25.85			56	64.52	22.08		
Completed university										
Duration of AR (years)										
<9	194	67.88	24.09	0.095		62	67.42	22.68	0.374	*Excluded*
≥9	190	63.67	25.57			39	61.89	25.91		
Etiologic allergen										
Dust mite	111	62.49	25.32	0.118		37	62.4	26.8	0.976	*6.757*
Pollen	190	66.68	25.27			41	63.9	21.27		
Both	75	67.51	25			21	75.72	18.44		
Familiar history of atopy										
Yes	214	64.81	26.59	0.689		68	66.11	23.72	0.766	*Excluded*
No	166	67.17	23.06			33	64.77	23.73		
Number of visits to allergist										
<3	196	66.07	24.76	0.888		57	65.44	22.73	0.898	*Excluded*
≥3	187	65.22	25.49			45	64.98	25.54		
Type of AR										
Intermittent	118	74.19	20.92	*0.000*	*−9.232*	36	71.01	19.02	0.123	*−10.012*
Persistent	268	61.98	25.9			65	62.5	25.75		
VALERO severity										
Mild	66	78.6	20.61	*0.000*	*−4.308*	14	75.68	23.63	0.079	*Excluded*
Moderate	205	63.84	24.7			64	65.65	22.17		
Severe	115	61.66	25.98			24	58.05	26.91		
His tory of allergy										
No	48	66.71	21.47	0.85		13	56.88	27.74	0.223	*Excluded*
Yes	338	65.57	25.6			89	66.46	23.2		
Type AIT										
SCIT	231	66.73	26.36	0.184		54	63.28	26.72	0.845	*Excluded*
SLIT	141	65.19	22.79			41	67.03	19.43		

“Excluded” are the factors discarded by the regression model as potentially associated with productivity impairment

Mean overall scores of the ESPIA questionnaire are obtained from the sum of its items with transformation to a 0–100-point scale

Significance values are in italics

*AIT* allergen immunotherapy, *AR* allergic rhinitis, *SD* standard deviation

* P-value for the bivariate analysis was calculated using Mann–Whitney or Kruskal–Wallis tests for independent samples, with a confidence interval of 95 %

^#^ In the population of adult students, all variables were included in the simple regression due to lack of significance in the initial bivariate analysis

## Discussion

For the first time in Spain and under “real life” conditions, the ENERGY study examined the impact of AR on work/academic performance and HRQOL of adult patients poorly controlled with or without pharmacological treatment. The study used the validated questionnaires WPAI + CIQ-AS, ESPRINT-15, and ESPIA, the tools specifically developed for allergies. Our results suggest that AR affects several functional areas. The data are in accord with the existing reports of the impairment of work/academic productivity [3, 4, 6] and HRQOL [7, 8] by AR in other countries. As could be expected, the negative impact of AR was more pronounced in patients with persistent and more severe forms of the disease, aspect already observed in a previous study conducted in Spain [15, 21]. The patients in these groups were less satisfied with their AIT than other patients.

The WPAI questionnaire has been used before to quantify the impact of several pathologies, such as irritable bowel disease, Crohn’s disease, and ankylosing spondylitis, on various aspects of productivity [22–24]. Here, absenteeism from workplace or classroom caused by AR (assessed by the WPAI + CIQ:AS) was relatively low. The work/class impairment (presenteeism) and overall loss of work/academic productivity were around 21 %. The affected patients might not perform well in their work or studies, their job- and class-related relationships might be damaged, and their professional growth might suffer. The participants also reported negative effects of AR outside work or study class; these effects are referred to as daily activity impairment. WPAI + CIQ:AS scores in this study were slightly lower than those published in previous reports. De la Hoz Caballer et al. [5] found an overall loss of work productivity of 26.8 %. However, they have used the generic WPAI questionnaire to compare the impact of different pathologies attended to in primary care centers. A Japanese study used WPAI-AS to evaluate the effect of oral fexofenadine on work productivity of seasonally allergic patients [25]. They reported an overall loss of productivity of 38.0 %. However, they did not include the mild AR patients and the study was conducted during pollen season [25]. AR symptoms are often experienced by the patients at work or at the academic center; thus, presenteeism is highly relevant to the evaluation of costs associated with AR [8]. Indeed, AR shares common pathophysiological components with asthma, otitis media, chronic sinusitis, upper respiratory infections, and nasal polyps [26, 27]. When these comorbidities are viewed as part of the continuum of AR disease, the overall cost of AR increases considerably [28, 29]. A population survey in Northern California has assessed the relative work loss and decrease in productivity during 4 weeks of AR and asthma in 400 adults. The results showed similar work loss levels for the two conditions, 23 % for AR and 24 % for asthma. However, among the symptomatic individuals who stayed at work, 36 % of patients with AR were less effective at their jobs in comparison with 19 % of asthma sufferers [3].

Impairment in HRQOL of AR patients has been a frequent object of study [8, 30]. Disease-specific questionnaires, such as the ESPRINT-15 tool, are the instruments most widely accepted for HRQOL evaluation. The ESPRINT-15 questionnaire accurately describes the problems most commonly associated with the disease. It includes the sleep loss caused by the AR symptoms, leading to fatigue and lack of concentration during the day, psychological effects, and deterioration in daily activities [8]. The present study showed that the increase in the persistence and severity of AR symptoms augmented the effect on patient HRQOL, similarly to previous results [21, 31]. The patients undergoing AIT reported better HRQOL than the individuals not receiving AIT. This finding confirms the reports that the severity of the AR has a stronger effect on the quality of life of patients than the duration of the disease [32].

These results justify the effort put into optimizing the allergy therapies. Pharmacological treatments are generally effective and well tolerated, improve sleep quality, reduce daytime fatigue [33, 34], and improve work productivity [35] and quality of life [30, 36]. However, the patients receiving such treatment often present poor levels of control of their nasal symptoms. Specific AIT can induce specific immune tolerance and has a long-term disease-modifying effect. Nevertheless, it is associated with low adherence [37], possibly because of a large number of administrations and the duration of the therapeutic course. The ESPIA questionnaire explores the pivotal aspects of subjective experience with AIT, such as self-perceived efficacy, daily life activities, cost–benefit balance, and general satisfaction. Importantly, the cost–benefit balance section assesses the compatibility of the daily “inconveniences,” caused by the logistics of the treatment administration, with the lifestyle of the patient. It makes possible to establish whether these inconveniences are compensated by AIT efficacy, which is a crucial determinant of adherence. Here, the overall satisfaction with AIT was high, especially for patients with intermittent and mild forms of AR, and independently of receiving SLIT or SCIT. According to their reports, most of the patients receiving AIT had reduced the use of pharmacological agents and were experiencing fewer symptoms since the initiation of the therapy. The regression analyses revealed that AIT was associated with decreased impact on work/academic productivity, on daily activities, and overall HRQOL. The ESPIA questionnaire and other patient-reported scales, along with existing efficacy assessment tools (symptom scores, current medication), may help to balance efficacy with tolerability in regular clinical practice. There are some limitations to the study to be considered. The inherent limitations in all observational and cross-sectional studies did not allow the assessment of the long-term effects. Despite that patients were included during May and October and most of them were allergic to pollens and/or HDM, meaning that they must be at their peak symptomatic seasons, we could not establish whether the results would hold true during the periods of high or low allergen counts. The patient selection comprised the whole range of AR severity, including mild and intermittent disease, which might have reduced the perceived impact on patient activity performance and HRQOL. The studied individuals were under different treatment regimens and their combinations. Some treatments might have caused somnolence, affecting the work/academic productivity and precluding the attribution of the observed impairment solely to AR symptoms. Indeed, in this study, sleep quality was only evaluated as an item of the ESPRINT-15 questionnaire. Furthermore, all the subjects in our study were under the care of an allergist and were continuing on SCIT or SLIT that they found beneficial. Therefore, it is not possible to make comparisons with patients who have failed or refused immunotherapy. Nevertheless, our results suggest a social impact on the overall AR population and should lead to larger, prospective studies with populations that are more specific. To reduce the socioeconomic impact of AR symptoms, we urgently need to determine the real burden of this common disease and find the most effective medical interventions.

## Conclusion

Our results showed a negative impact of AR on work/academic productivity and HRQOL of patients receiving pharmacological treatment in several allergy departments in Spain. Several factors such as persistent AR or a more severe form of the disease were associated with higher impairment of the studied functional outcomes. Specific AIT may play a protective role, improving productivity and HRQOL of AR patients. A more comprehensive interaction between patient and physician might be required to reduce the current socioeconomic burden of this disease. Various parameters including nature, severity, and impact of AR symptoms on self-perceived health-related measures should be taken into account.

## Abbreviations

AIT: allergen immunotherapy
AR: allergic rhinitis
ESPIA: Satisfaction Scale for Patients Receiving Allergen Immunotherapy (from Spanish)
HRQOL: health-related quality of life
SD: standard deviation
SLIT: sublingual immunotherapy
WPAI + CIQ:AS: Work Productivity and Activity Impairment Questionnaire and Classroom Impairment Questions: Allergy Specific
